# Gut Microbiome Profiles Are Associated With Type 2 Diabetes in Urban Africans

**DOI:** 10.3389/fcimb.2020.00063

**Published:** 2020-02-25

**Authors:** Ayo P. Doumatey, Adebowale Adeyemo, Jie Zhou, Lin Lei, Sally N. Adebamowo, Clement Adebamowo, Charles N. Rotimi

**Affiliations:** ^1^Center for Research on Genomics and Global Health, National Human Genome Research Institute, National Institutes of Health, Bethesda, MD, United States; ^2^Department of Epidemiology and Public Health and Greenebaum Comprehensive Cancer Center, University of Maryland School of Medicine, Baltimore, MD, United States; ^3^Center for Bioethics and Research, Ibadan, Nigeria; ^4^Institute of Human Virology, University of Maryland School of Medicine, Baltimore, MD, United States

**Keywords:** gut microbiome, type 2 diabetes, urban Africans, 16S V4 rRNA sequencing, microbial composition

## Abstract

Gut dysbiosis has been associated with several disease outcomes including diabetes in human populations. Currently, there are no studies of the gut microbiome composition in relation to type 2 diabetes (T2D) in Africans. Here, we describe the profile of the gut microbiome in non-diabetic adults (controls) and investigate the association between gut microbiota and T2D in urban West Africans. Gut microbiota composition was determined in 291 Nigerians (98 cases, 193 controls) using fecal 16S V4 rRNA gene sequencing done on the Illumina MiSeq platform. Data analysis of operational taxonomic units (OTU) was conducted to describe microbiome composition and identify differences between T2D and controls. The most abundant phyla were *Firmicutes, Actinobacteria*, and *Bacteroidetes*. *Clostridiaceae*, and *Peptostreptococcaceaea* were significantly lower in cases than controls (*p* < 0.001). Feature selection analysis identified a panel of 18 OTUs enriched in cases that included *Desulfovibrio piger, Prevotella, Peptostreptococcus, and Eubacterium*. A panel of 17 OTUs that was enriched in the controls included *Collinsella, Ruminococcus lactaris, Anaerostipes*, and *Clostridium*. OTUs with strain-level annotation showing the largest fold-change included *Cellulosilyticum ruminicola* (log_2_FC = −3.1; *p* = 4.2 × 10^−5^), *Clostridium paraputrificum* (log_2_FC = −2.5; *p* = 0.005), *and Clostridium butyricum* (log_2_FC = −1.76; *p* = 0.01), all lower in cases. These findings are notable because supplementation with *Clostridium butyricum* and *Desulfovibrio piger* has been shown to improve hyperglycemia and reduce insulin resistance in murine models. This first investigation of gut microbiome and diabetes in urban Africans shows that T2D is associated with compositional changes in gut microbiota highlighting the possibility of developing strategies to improve glucose control by modifying bacterial composition in the gut.

## Introduction

The gut microbiota (GM) has been intensely studied over the past few years for its involvement in health and disease (Forslund et al., 2015; Kataoka, 2016; Lynch and Pedersen, 2016; Tang et al., 2017). These investigations have provided novel insights into the important role that GM plays in host nutrition, metabolism, and immunity (Komaroff, 2017; Pascale et al., 2018). GM produces numerous biochemical molecules including vitamins, amino-acids, and short chain fatty acids that are involved in the proper functioning of human organs and systems. It has been suggested that the GM function as an additional endocrine system (Pascale et al., 2018). The composition of GM is considerably influenced by factors such as lifestyle, age, seasonal variations, and geography (De Filippo et al., 2010, 2017; Yatsunenko et al., 2012; Schnorr et al., 2014). Comparative studies have shown that human GM from industrialized societies e.g., North America and Europe have different gastro-intestinal microbial profiles compared to what is observed in less industrialized societies e.g., South America and Africa (De Filippo et al., 2010, 2017; Yatsunenko et al., 2012; Schnorr et al., 2014). Indeed, a recent study showed that migration from a non-Western country to the United States of America (USA) was associated with loss of gut microbiome diversity and function. The USA-associated strains and functions displace native strains and functions, and these effects increase with age and duration of US residence (Vangay et al., 2018).

Although GM has been associated with many pathologies including type 2 diabetes (T2D), atherosclerosis, inflammatory bowel disease, and cancer (Lynch and Pedersen, 2016; Komaroff, 2017, 2018), most of these studies were conducted in westernized societies or in animal models (Zhang et al., 2010; Bech-Nielsen et al., 2012; Qin et al., 2012; Forslund et al., 2015; Kataoka, 2016). To date, only a handful of studies have investigated the composition of gut microbiome or its relationship to disease and health in Africans (Grześkowiak et al., 2012; Yatsunenko et al., 2012; Schnorr et al., 2014; Morton et al., 2015; Cheung et al., 2016; Iebba et al., 2016; Davis et al., 2017; De Filippo et al., 2017; Hansen et al., 2019) (Table 1). Furthermore, these studies conducted in Africans have focused on description of GM, relationship between GM and childhood malnutrition, lifestyles, environmental adaptation, or presence of other parasites (De Filippo et al., 2010, 2017; Yatsunenko et al., 2012; Schnorr et al., 2014; Morton et al., 2015) (Table 1). While some of these studies demonstrate differences in GM composition between African populations and between African and European or American groups for example (Schnorr et al., 2014), little is known about the role of GM in relation to diseases, especially metabolic disorders such as obesity and T2D in Africans (Upadhyaya and Banerjee, 2015). The current paucity of data in Africans is concerning given the well-established associations between GM and several disease outcomes in populations of European ancestry as well as in animal models (Liu et al., 2018; Weickert and Pfeiffer, 2018). Reported specific associations include decreased abundance in *Akkermansia muciniphila* (Zhang et al., 2013) and butyrate-producing bacteria (Brunkwall and Orho-Melander, 2017) in diabetes and association between insulin resistance and branch-chain amino-acid (BCAA)-producing species such as *Prevotella copri* and *Bacteroides vulgaris* (Wang et al., 2011; Pedersen et al., 2016). Furthermore, the increasing global prevalence of cardiometabolic diseases such as T2D in low- and middle-income countries (LMIC) calls for investigations that go beyond descriptions of GM, to those that assess relationships between GM and disease outcomes in these societies where critical data are currently lacking.

**Table 1:** List of microbiome studies conducted in Sub-Saharan Africa.

**Study**	**Year**	**Region/country**	**Population**	**Design**	**References**
Population structure of human gut bacteria in a diverse cohort from rural Tanzania and Botswana	2019	Tanzania and Botswana	Adults/Rural Hunter-Gathers, pastoralists, agropastoralists, Mixed hunter-gathers/agropastoralist	Comparative study (geographic and subsistence lifestyle)	Hansen et al., 2019
Diet, environments, and gut microbiota. A preliminary investigation in children living in rural and Urban Burkina Faso and Italy	2017	Burkina Faso	Children/rural and urban	Comparative study (within the same country)	De Filippo et al., 2017
Atopic dermatitis and food sensitization in South African toddlers: role of fiber and gut microbiota	2017	South Africa	Children/Urban	Comparative study (disease state and controls)	Mahdavinia et al., 2017
Seasonal cycling in the gut microbiome of the Hadza hunter-gatherers of Tanzania	2017	Tanzania	Children and adults, Hunter-gatherers age > 3	Comparative study (dry season vs. wet season	Smits et al., 2017
Variation in Rural African Gut microbiota is strongly correlated with colonization by entamoeba and subsistence	2015	Cameroon	Adults/ Hunter-gathers, farmers, fishermen	Comparative study (mode of subsistence in same environment and degree of urbanization)	Morton et al., 2015
Metagenome Sequencing of the Hadza Hunter-gatherer gut microbiota	2015	Tanzania	Adults and children/hunter-gathers	Comparative functional Analysis, gut microbiome resistome profile	Rampelli et al., 2015
Gut microbiome of the Hadza hunter-gatherers	2014	Tanzania	Adults and children/hunter-gathers	Descriptive and comparative study across different populations (including mode of subsistence)	Schnorr et al., 2014
Human gut microbiome viewed across age and geography	2012	Malawi	Adults and children/monozygotic and dizygotic twin pairs/Rural	Comparative study across socio-geographic populations and age range	Yatsunenko et al., 2012
Impact of diet in shaping gut microbiota revealed by a comparative study in children from Europe and rural Africa	2010	Burkina Faso	Children/rural and urban	Comparative study (inter-continental)	De Filippo et al., 2010

Here, we provide an analysis of the GM in 291 unrelated adults enrolled from an urban center in Nigeria with the aims of describing the GM microbial composition in non-T2D population samples (controls) and comparing the phylogenetic diversity and taxonomic relative abundance between controls and cases. We also conducted functional analyses to predict genes and pathways abundance in these two groups. To our knowledge, this is the first investigation of the association between GM and T2D in Africa and is so far the largest study of GM conducted in Africa populations.

## Materials and Methods

### Study Participants

Included in this investigation are 291 participants (193 controls and 98 cases) from the longstanding genetic epidemiology study of T2D in Africa—the Africa America Diabetes Mellitus (AADM) study—which has been previously described elsewhere (Rotimi et al., 2001; Adeyemo et al., 2015). Participants for the microbiome studies were enrolled at a single site—Ibadan, Nigeria—one of the largest cities in sub-Saharan Africa (SSA). Briefly, ethical approval was obtained from the Institutional Review Board of the participating institution. Written informed consent was obtained from all participants. Demographic information was collected using standardized questionnaires and anthropometric, medical history, and clinical examination parameters were obtained by trained study staff during a clinic visit. Weight was measured in light clothes on an electronic scale to the nearest 0.1 kg and height was measured with a stadiometer to the nearest 0.1 cm. Body mass index (BMI) was computed as weight (kg) divided by the square of height in meters (m^2^). All serum biochemistry (fasting glucose, insulin, total cholesterol, HDL-cholesterol, LDL-cholesterol, and triglycerides) were measured using a COBAS® Integra Analyzer Series (Roche Diagnostics, Indianapolis, Indiana). The definition of T2D was based on the American Diabetes Association (ADA) criteria: a fasting plasma glucose concentration (FPG) ≥ 126 mg/dl (7.0 mmol/l) or a 2-h postload value in the oral glucose tolerance test ≥ 200 mg/dl (11.1 mmol/l) on more than one occasion. Alternatively, a diagnosis of T2D was accepted if an individual was on pharmacological treatment for T2D and review of clinical records indicated adequate justification for that therapy. The detection of autoantibodies to glutamic acid decarboxylase (GAD) and/or a fasting C-peptide ≤ 0.03 nmol/l was used to exclude probable cases of type 1 diabetes. Controls were required to have FPG < 126 mg/dl or 2-h post load of < 140 mg/dl and no symptoms suggestive of diabetes (the classical symptoms being polyuria, polydipsia, and unexplained weight loss).

Fecal samples were collected from each consenting participant during a clinic visit and frozen at −80°C until shipped by expressed courier to our lab at the National Institutes of Health (NIH) in the USA where the samples remained frozen until processed for DNA extraction.

### DNA Extraction From Fecal Samples

DNA extraction was performed with the MoBioPowerMag® Microbiome kit (Carlsbad, CA) according to the manufacturer's guidelines and optimized for high-throughput processing. All samples were quantified using the Qubit® Quant-iT dsDNA High Sensitivity Kit (Invitrogen, Life Technologies, Grand Island, NY) to ensure that they met minimum concentration and mass of DNA.

### Library Preparation and Profiling

Samples were enriched for bacterial 16S V4 rDNA region, by amplifying the DNA samples utilizing fusion primers designed against the surrounding conserved regions which are tailed with sequences to incorporate adapters and indexing barcodes (Illumina, San Diego, CA). Each sample was PCR amplified with two differently bar coded V4 fusion primers and PCR products were quantified by fluorometric method (Qubit or PicoGreen from Invitrogen, Life Technologies, Grand Island, NY). Samples that met the post-PCR quantification minimum were pooled equimolar and sequenced.

A pool containing 16S V4 enriched, amplified, barcoded samples was loaded into a MiSeq® reagent cartridge, and then onto the instrument along with the flow cell. After cluster formation on the MiSeq instrument, the amplicons were sequenced for 250 cycles with custom primers designed for paired-end sequencing. Quality control (QC) and quality assurance (QA) metrics are maintained for all sample handling, processing, and storage procedures.

### Bioinformatic Analysis and Statistical Methods

The data analysis pipeline for microbial profiling was done using SecondGenomeR package 2.2.0 which incorporates the steps of pre-processing, summarization, normalization, alpha-diversity metrics (within sample diversity), beta diversity metrics (inter-sample similarity), ordination/clustering, sample classification, and significance testing. A Second Genome Solutions' proprietary software package as well as MicrobiomeAnalyst, a web-based pipeline, were used for statistical analyses and visual exploration (Dhariwal et al., 2017).

#### Operation Taxonomic Unit (OTU) Selection and Summarization

Sequenced paired-end reads were merged using USEARCH and the resulting sequences were compared to an in-house strains database (http://www.secondgenome.com/solutions/resources/data-analysis-tools/strainselect/) using USEARCH (usearch_global). All sequences matching a unique strain with an identity >=99% were assigned a strain OTU. To ensure specificity of the strain hits, a difference of >=0.25% between the identity of the best hit and the second-best hit was required (e.g., 99.75 vs. 99.5). For each strain OTU, one of the matching reads was selected as representative and all sequences were mapped by USEARCH (usearch_global) against the strain OTU representatives to calculate strain abundances. The remaining non-strain sequences were quality filtered and dereplicated with USEARCH. Samples with <50,000 reads were removed. Resulting unique sequences were then clustered at 97% by UPARSE (*de novo* OTU clustering) and a representative consensus sequence per *de novo* OTU was determined (Edgar, 2013). The UPARSE clustering algorithm comprises a chimera filtering and discards likely chimeric OTUs. All non-strain sequences that passed the quality filtering were mapped to the representative consensus sequences to generate an abundance table for *de novo* OTUs. Representative OTU sequences were assigned taxonomic classification via mothur's bayesian classifier, trained against the Greengenes reference database of 16S rRNA gene sequences (greegenes.lbl.gov) clustered at 99% similarity (Mcdonald et al., 2012).

Independent filtering reduced the number of OTUs from 3,229 to 1,165 and the number of sequences from 51,959,463 to 51,655,653 (Figure S1). Approximatively 99% of the sequences were classified at phylum, class, and order levels, whereas 93.2% were classified at family, 52.2% at genus level, 26% at species and 24% at strain levels (Figure S2). The rarefaction curve approached plateau indicating near-completeness of the captured microbiome profiles (Figure S3).

Following taxa identification, the values used for each taxa-sample intersection were populated with the abundance of reads assigned to each OTU in an “OTU table.” A corresponding table of OTU Greengenes classification was also generated. The *Phyloseq* R package was used to analyze generated metadata, taxonomy, and sequence counts (Mcmurdie and Holmes, 2013).

#### Diversity Metrics, Ordination, Clustering, and Classification Methods

Alpha-diversity, a measure of within sample diversity, was computed using two metrics: OTU richness and Shannon diversity (Ce, 1948). Similarly, beta-diversity (a measure of microbial community compositional differences between samples) was computed using the Bray-Curtis dissimilarity metric (Bray and Curtis, 1957). Permutational Analysis of Variance (PERMANOVA) was used to access significant differences between cases and controls. In this randomization/Monte Carlo permutation test, the samples are randomly reassigned to the various sample categories, and the expected between-category differences are compared to the observed between-category differences. PERMANOVA utilizes the sample-to-sample distance matrix directly, not a derived ordination or clustering outcome. Kruskal-Wallis rank sum test was used to compare GM structure between cases and controls for alpha and beta diversity and proportional abundances, which are reported as mean percentage relative abundance unless specified. Two-dimensional ordinations Principal Coordinate Analysis (PCoA) and hierarchical clustering maps (Ward's method) of the samples were generated in the forms of dendrograms to visually summarized the inter-sample relationships.

#### Metagenomic Inference

Piphillin, an algorithm independent of phylogenetic tree that leverages the most contemporary functional genome databases (e.g., KEGG 70.1), was used to estimate the functional capacity of the GM in cases and controls (Iwai et al., 2016). A genome was inferred for each 16S rRNA OTU based on the sequence identity between an OTU's representative sequence and the nearest neighbor 16S rRNA sequence from the genome databases restricted to a minimum identity of 97%. OTU abundance was normalized by 16s rRNA copy numbers and then multiplied by the gene contents of each inferred genome to predict each sample's metagenome.

The *DESeq2* package was used to evaluate univariate differential abundance of OTUs, genes and pathways as previously described (Love et al., 2014; Mcmurdie and Holmes, 2014). A negative binomial noise model for the over-dispersion and Poisson process intrinsic were applied to generate data thus accounting for both technical and biological variability between the experimental conditions. We implemented *DESeq2* using the default settings and q-values were calculated using the Benjamini-Hochberg procedure to correct p-values by controlling for false discovery rates (Benjamini and Hochberg, 1995). An OTU is considered differentially abundant if adjusted p-values were <0.05 and the absolute value of the logFC ≥ 1.

## Results

Consistent with expectations, several anthropometric, and clinical parameters were significantly different between cases and controls. Compared to controls, cases had higher BMI (32 vs. 30 Kg/m^2^) and larger waist circumference (102.0 vs. 97 cm). Cases also had unfavorable lipid and glycemic profiles with fasting glucose, insulin, HOMA-IR, hemoglobin A1C, and triglycerides levels being much higher in cases than in controls, whereas HDL-C was lower (Table 2). Nearly all cases (97%) in this study were on treatment, with 38.8% on metformin (Met) only, 5.1% on sulfonylurea (SU) only, 49% on metformin and sulfonylurea (Met+SU), and 4.1% on combinations of anti-diabetes medication. Three cases (3.1%) were treatment-naïve.

**Table 2:** Characteristics of gut microbiome study participants: the AADM study.

**Characteristic**	**Controls (*N* = 193)** **Mean (SD)**	**Cases (*N* = 98)** **Mean (SD)**	*P* value^*^
Age (years)	54.3 (13.3)	59.7 (10.4)	**0.0002**
Body mass index (BMI, kg/m^2^)	30.33 (6.09)	32.21 (5.94)	**0.0125**
Percent fat mass (PFM, %)	37.30 (9.65)	39.62 (10.62)	0.0641
Waist circumference (cm)	97.11 (12.56)	101.83 (12.34)	**0.0025**
Waist-to-hip ratio (WHR)	0.94 (0.08)	0.96 (0.09)	0.0549
Glucose (mg/dl) ^¥^	82.0 (76–89)	118 (96–157)	**<0.0001**
Insulin (uIU/ml)^¥^	7.2 (4.4–10.5)	9.2 (5.8–13.0)	**0.0022**
HOMA-IR^¥^	1.51 (0.87–2.20)	2.67 (1.76–4.68)	**<0.0001**
HbA1C (%) ^¥^	5.40 (5.2–5.7)	7.4 (6.20–8.80)	**<0.0001**
Total cholesterol (mg/dl)	193.20 (51.34)	201.16 (64.12)	0.288
HDL-cholesterol (mg/dl)^¥^	24 (13–37)	31 (20–44)	**0.0272**
LDL-cholesterol (mg/dl)	123.10 (44.08)	122.72 (49.77)	0.9478
Triglycerides (mg/dl)	94.82 (41.51)	120.28 (59.57)	**0.0002**

*Significantly different between cases and controls at p < 0.05 in bold*.

* *P-value for t-test except for variables with for Wilcoxon rank-sums test was used*.

¥ *indicates median (interquartile range). All other figures are mean (SD)*.

### Gut Microbiome Composition in Controls

**Figure 1:**
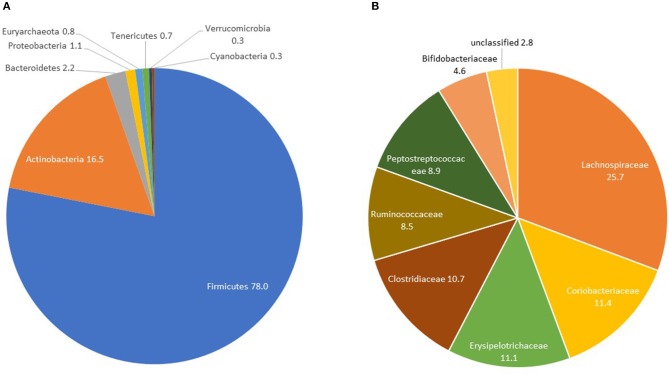
GM composition in controls at the phylum **(A)** and family **(B)** levels: the AADM study.

Controls had a median OTU richness of 448 (IQR 377-519) and median Shannon Diversity of 3.45 (IQR 3.14-3.70). Shannon Diversity and OTU richness were not significantly associated with age, gender or measures of body composition (BMI, waist circumference and percent fat mass)—(Table S1). The only covariate significantly associated with beta-diversity was gender (*p* = 0.032)—Table S1. At the phylum level, Firmicutes constituted the majority with percent relative abundance of 78% (median 80.07%) followed by Actinobacteria (mean: 16.5%, median: 14.8%) and Bacteroidetes (mean: 2.2%, median: 0.3%) (Figure 1A and Table 3). At the family level, the most represented taxa were *Lachnospiraceae* (25.7%), *Coriobacteriaceae* (11.4%), *Erysipelotrichaceae* (11.1%), *Clostridiaceae* (10.7%), and *Peptostreptococcaceae* (8.9%) (Figure 1B). *Blautia* (15%) represented the most abundant genus followed by *Collinsella* (8%), *Ruminococcus* and *Bifidobacterium* (5%). Other genera including Catenibacterium, *Eubacterium, Streptococcus, Prevotella* were identified with relative abundances of <5% (Figure S6).

**Table 3:** Comparative relative abundances of the gut microbiome's most abundant phyla in controls and cases: the AADM study.

**Taxonomic Rank/OTU**	**Median (Controls)**	**IQR (Controls)**	**Median (Cases)**	**IQR (Cases)**	Chi-square^*^	***P***
**Phylum**						
Firmicutes	80.07	71.18, 86.03	76.61	69.51, 83.33	5.759	**0.0164**
Actinobacteria	14.80	9.25, 22.32	16.66	10.57, 23.62	2.432	0.1189
Bacteroidetes	0.30	0.09, 1.07	0.44	0.18, 1.38	3.861	**0.0494**
Proteobacteria	0.13	0.03, 0.60	0.18	0.05, 0.85	2.053	0.1519
Euryarchaeota	0.02	0.00, 0.98	0.15	0.00, 2.29	3.876	**0.049**
Tenericutes	0.47	0.09, 1.07	0.78	0.14, 1.36	3.703	0.0543
Verrucomicrobia	0.00	0.00, 0.02	0.00	0.00, 0.02	0.008	0.9273
Cyanobacteria	0.04	0.00, 0.25	0.04	0.00, 0.28	0.05	0.8227
**Family**						
Lachnospiraceae	25.01	19.39, 31.72	24.16	17.60, 31.56	0.315	0.5744
Coriobacteriaceae	10.95	6.54, 14.97	12.12	6.93, 16.25	1.19	0.2754
Erysipelotrichaceae	9.27	4.48, 15.88	10.71	5.58, 15.57	0.458	0.4987
Clostridiaceae	8.69	3.61, 15.73	5.50	1.14, 12.14	9.83	**0.0017**
Ruminococcaceae	6.65	3.62, 11.39	7.63	3.94, 13.58	2.596	0.1072
Peptostreptococcaceae	7.56	3.30, 13.53	4.62	0.77, 10.15	12.704	**0.0004**
Bifidobacteriaceae	0.28	0.02, 5.27	1.04	0.02, 8.16	1.049	0.3056
Unclassified	2.68	1.79, 3.63	2.56	1.75, 3.72	0.168	0.682

*Values are median (interquartile range) of percentage relative abundance means*.

* *Kruskal-Wallis H test*.

*P < 0.05 in bold*.

### Diversity Analysis

**Figure 2:**
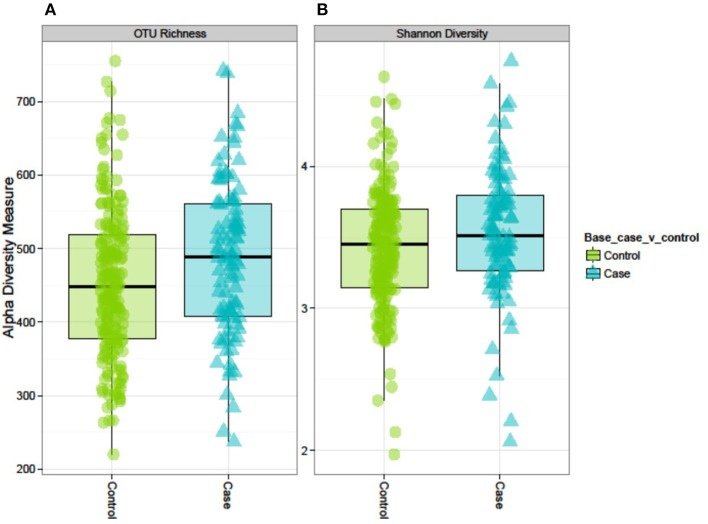
Alpha diversity of the GM in controls and cases: the AADM study. **(A)** OTU richness represents the number of OTUs present in each sample. **(B)** Shannon diversity index accounts for the richness and evenness of OTUs within a sample.

Both alpha-diversity indices used in this study were significantly higher in cases than in controls (Figure 2). The median (IQR) OTU richness was significantly different between cases 489 (408–561) and controls 448 (377–519), *p* = 0.008. Similarly, the median (IQR) Shannon Diversity was 3.52 (3.26–3.80) vs. 3.45 (IQR 31.4–3.70), respectively in cases and controls (*p* = 0.04). However, we did not observe any distinct clustering of microbial communities between cases and controls as shown by the PCoA plot or Ward's method (Figure S5). PERMANOVA analysis of the Bray-Curtis dissimilarity matrix (an index of beta-diversity) demonstrated that T2D is strongly associated with microbiome abundance profile as measured by beta diversity [*F*_(1, 290)_ = 2.55, *p* < 0.001].

### *Firmicutes* Are Significantly Decreased Among Diabetics in This Study of Sub-Saharan Africans

**Figure 3:**
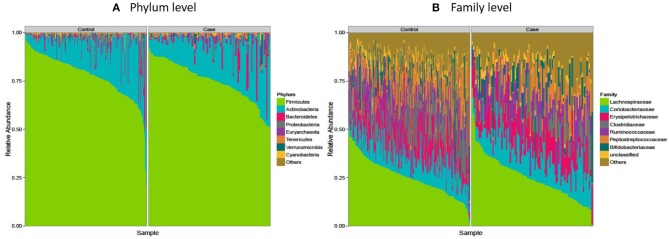
Microbiota composition in controls and cases: the AADM study. **(A)** Shows microbial composition at the phylum level while **(B)** shows microbial composition at the family level. The most abundant taxa are labeled.

The microbiota composition in cases displayed a significantly different profile at both phylum and family levels when compared to that of the controls. *Firmicutes*, the largest microbial community was moderately decreased in cases compared to controls (median: 76.6 vs. 80%, *p* = 0.02) and correspondingly an increase in *Actinobacteria* (mainly *Coriobacteriaceae* and *Bifidobactericeae*—median: 16.7%), while *Bacteroidetes* (mainly *Prevotella*), *Euryarchaeota*, and *Tenericutes* were nominally increased in cases (*p* = 0.05) (Figure 3A and Table 3). At the family level, Clostridiaceae and *Peptostreptococcaceae* were significantly decreased in cases compared to controls (*p* < 0.05) while no significant difference was observed among other families of the Firmicutes phylum (*Lachnospiraceae, Erysipelotrichaceae, Bifidobacteriaceae*) (Figure 3B and Table 3).

### Effect of Treatment on Gut Microbiota in Diabetes

Median Shannon Diversity (a measure of α-diversity) was higher in the treatment groups (Met only, Met+ SU, SU only, other combinations) compared to controls indicating a treatment effect (Figure S4), while the untreated cases seemed to have the lowest α-diversity and higher *Firmicutes* (86%) compared to all the treatment classes. However, their numbers are too few (only 3 cases) to draw meaningful conclusions. The average percent relative abundance of Bacteroidetes is the same across the different groups (~2%) except for “other combinations” group (*Bacteroidetes*, 7%) (Figure S7).

Metformin is the most widely used treatment in T2D and had been studied in other populations. To assess its effect on the relative abundance of key taxa, we utilized regression models (either non-parametric regression or Poisson regression depending on the data distribution) in the diabetic subset with and without adjusting for age, sex, and BMI as covariates. Metformin treatment seems to be associated with lower relative abundance of *Firmicutes* (*p* = 0.004) in the unadjusted model. However, the effect was nullified after adjustment for covariates (Table S2A). At the family level, the relative abundance of *Verrucomicrobiaceae* appeared to increase with metformin treatment (beta = 2.14, *p* = 0.03) in the unadjusted model, but the model was not significant after adjustment (*p* = 0.08). No apparent effect of metformin was observed on other microbial families (Table S2B).

### Differentially Abundant Microbial Features in Diabetes

**Figure 4:**
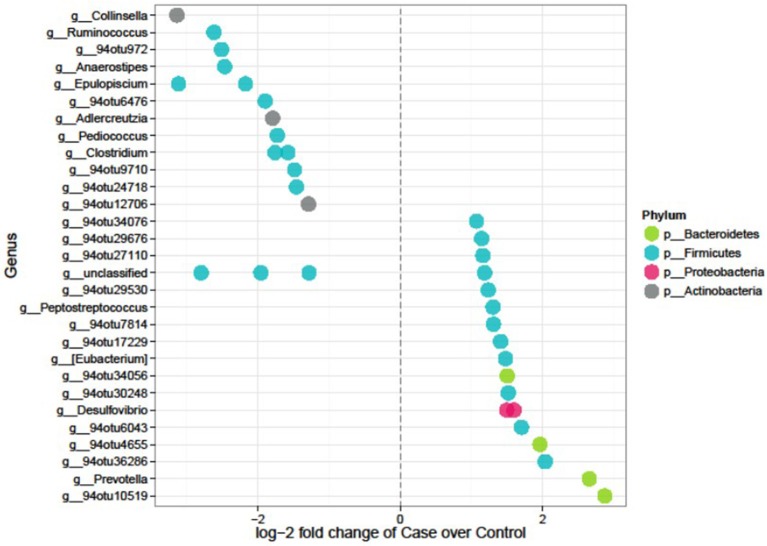
Differentially abundant features in cases vs. controls in the AADM study. Each point represents an OTU belonging to each genus. Points are color-coded by phylum.

Feature selection identified 35 significant differences out of the 1,165 tested features. Seventeen OTUs were lower whereas 18 were higher in cases compared to controls (Figure 4 and Table 4). The majority (~74%) of the OTUs differentially abundant in diabetes belongs to the *Firmicutes* and includes genera such as *Anaerostipes* (log2FC = −2.5, *p* = 4.01 × 10^−5^), *Ruminococcus* (logFC = −2.62, *p* = 4.0 × 10^−5^), *Clostridium* (logFC = −1.8, *p* = 0.01), and *Epulopiscium* (logFC = −2.2, *p* = 0.02) that were less abundant in cases compared to controls whereas *Peptostreptococcus* (logFC = 1.3, *p* = 0.040), and *Eubacterium* (logFC = 1.48, *p* = 0.048) were more abundant in cases. *Prevotella*, a member of *Bacteroidetes* was significantly abundant in cases (LogFC = 2.7, *p* = 4.5 × 10^−5^). Six of the 35 differentially abundant features had a strain-level annotation and half of them (*Cellulosilyticum ruminicola, Clostridium paraputrificum*, and *Clostridium butyricum* had large fold changes and were lower in cases (Table 4).

**Table 4:** Feature selection analysis for gut microbiome in cases vs. controls: the AADM study.

**Phylum**	**Family**	**Genus**	**Strain**	**log_**2**_FC**	***P***	**Adjusted *P* value**
Actinobacteria	Coriobacteriaceae	Collinsella	Unclassified	−3.14	1.84 × 10^−7^	4.01 × 10^−5^
Actinobacteria	Coriobacteriaceae	94otu12706	Unclassified	−1.29	0.002	0.04
Actinobacteria	Coriobacteriaceae	_Adlercreutzia	Unclassified	−1.79	9.7 × 10^−6^	0.0008
Firmicutes	Lachnospiraceae	Anaerostipes	Unclassified	−2.46	2.0 × 10^−7^	4.01 × 10^−5^
Firmicutes	Lachnospiraceae	Epulopiscium	Unclassified	−2.17	0.0008	0.02
Firmicutes	**Lachnospiraceae**	**Epulopiscium**	**(Cellulosilyticum ruminicola)**^**#**^	–**3.11**	**2.7 × 10**^**−7**^	**4.2** × **10**^**−5**^
Firmicutes	Lachnospiraceae	94otu29676	Unclassified	1.14	0.0010	0.025
Firmicutes	**Lachnospiraceae**	**94otu9710**	**(Ruminococcus lactaris ATCC 29176)**	–**1.49**	**0.0003**	**0.01**
Firmicutes	Peptostreptococcaceae	94otu24718	Unclassified	−1.46	0.0006	0.02
Firmicutes	Peptostreptococcaceae	Peptostreptococcus	Unclassified	1.30	0.002	0.04
Firmicutes	**Peptostreptococcaceae**	**Unclassified**	**([Clostridium] glycolicum)**	–**1.28**	**1.4 × 10**^**−5**^	**0.001**
Firmicutes	Clostridiaceae	Clostridium	unclassified	−1.58	5.4 × 10^−6^	0.0005
Firmicutes	**Clostridiaceae**	**Clostridium**	**(Clostridium butyricum)**^**#**^	–**1.76**	**0.0004**	**0.014**
Firmicutes	**Clostridiaceae**	**94otu972**	**(Clostridium paraputrificum)**^**#**^	–**2.51**	**8.3 × 10**^**−5**^	**0.005**
Firmicutes	Clostridiaceae	unclassified	Unclassified	−1.96	5.3 × 10^−5^	0.003
Firmicutes	unclassified	unclassified	Unclassified	−2.80	0.0001	0.007
Firmicutes	Ruminococcaceae	unclassified	Unclassified	1.19	0.0002	0.011
Firmicutes	Ruminococcaceae	Ruminococcus	Unclassified	−2.62	2.1 × 10^−7^	4.01 × 10^−5^
Firmicutes	Ruminococcaceae	94otu17229	Unclassified	1.41	8.1 × 10^−5^	0.005
Firmicutes	Ruminococcaceae	94otu6476	Unclassified	−1.90	0.002	0.04
Firmicutes	Ruminococcaceae	94otu27110	Unclassified	1.16	0.0009	0.02
Firmicutes	Ruminococcaceae	94otu6043	Unclassified	1.70	5.4 × 10^−5^	0.003
Firmicutes	Ruminococcaceae	94otu34076	Unclassified	1.07	0.0005	0.01
Firmicutes	Christensenellaceae	94otu29530	Unclassified	1.24	0.0004	0.01
Firmicutes	91otu17987	94otu36286	Unclassified	2.04	0.0004	0.01
Firmicutes	91otu8397	94otu30248	Unclassified	1.52	3.4 × 10^−6^	0.0004
Firmicutes	91otu9176	94otu7814	Unclassified	1.31	0.0010	0.03
Firmicutes	Erysipelotrichaceae	Eubacterium	Unclassified	1.48	0.002	0.045
Firmicutes	Lactobacillaceae	Pediococcus	Unclassified	−1.72	0.0007	0.02
Proteobacteria	Desulfovibrionaceae	Desulfovibrio	Unclassified	1.60	0.0004	0.01
Proteobacteria	**Desulfovibrionaceae**	**Desulfovibrio**	**(Desulfovibrio piger ATCC 29098)**	**1.50**	**0.0019**	**0.04**
Bacteroidetes	Prevotellaceae	Prevotella	Unclassified	2.66	3.5 × 10^−7^	4.5 × 10^−5^
Bacteroidetes	Paraprevotellaceae	g__94otu4655	Unclassified	1.96	0.0011	0.03
Bacteroidetes	91otu4650	g__94otu10519	Unclassified	2.87	2.2 × 10^−8^	1.7 × 10^−5^
Bacteroidetes	f__Rikenellaceae	g__94otu34056	Unclassified	1.50	0.0017	0.04

*In bold are shown the 6 significantly different OTUs with strain level annotation*.

# *Annotates OTUs with the largest fold change (FC) and lower in cases compared to controls*.

### Functional Profiles of Controls and Diabetes Gut Microbiome

**Figure 5:**
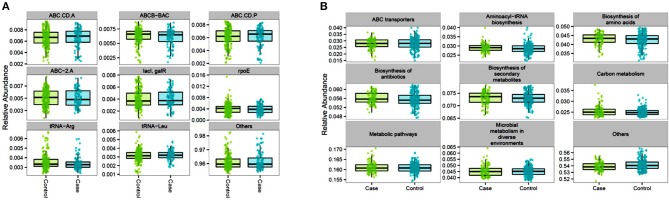
Proportional abundance of the top inferred genes **(A)** and pathways **(B)** for controls and cases gut microbiome: the AADM study.

Functional contributions of GM in cases and controls were explored based on OTUs using Piphillin. The results revealed 7,474 genes across all samples. The top most abundant functional genes are presented in Figure 5 and included several members of the ABC transport system ATP-binding proteins (ABC-2. A, ABC.CD.A, ABC.CD.P), LacI family transcriptional regulator (lacI, galR), and tRNA (tRNA-Arg and tRNA-Leu). While most of the abundant functional genes tended to be slightly higher in controls, there was no significantly different inferred genes between cases and controls (Table S3). Feature selection analysis on the inferred genes between cases and controls identified 16 genes (log 2 FC ≥1, *p* < 0.05) that were enriched in cases including Aspartate dehydrogenase (nadX) and cobaltochelatase (cobN) (Table 5 and Figure S8). However, after FDR adjustment they were no longer statistically significant.

**Table 5:** Feature selection analysis for inferred genes in cases vs. controls: list of 16 genes with significant unadjusted *p* < 0.05 and log_2_FC≥1.

**KO**	**Gene**	**log_**2**_FC**	**Unadjusted *p*-value**
K07033	Uncharacterized protein	1.34	7.3 × 10^−5^
K06989	nadX, ASPDH; aspartate dehydrogenase [EC:1.4.1.21]	1.27	0.010
K02230	cobN; cobaltochelatase CobN [EC:6.6.1.2]	1.22	0.007
K01802	peptidylprolyl isomerase [EC:5.2.1.8]	1.13	0.008
K16323	yxjA, nupG; purine nucleoside transport protein	1.12	0.002
K01858	INO1, ISYNA1; myo-inositol-1-phosphate synthase [EC:5.5.1.4]	1.11	0.013
K00641	metX; homoserine O-acetyltransferase/O-succinyltransferase [EC:2.3.1.31 2.3.1.46]	1.09	0.001
K01278	DPP4, CD26; dipeptidyl-peptidase 4 [EC:3.4.14.5]	1.08	0.042
K03658	helD; DNA helicase IV [EC:3.6.4.12]	1.07	0.014
K09005	Uncharacterized protein	1.07	0.002
K02977	RP-S27Ae, RPS27A; small subunit ribosomal protein S27Ae	1.06	0.013
K02303	cobA; uroporphyrin-III C-methyltransferase [EC:2.1.1.107]	1.06	0.004
K11031	slo; thiol-activated cytolysin	1.05	0.006
K02076	zurR, zur; Fur family transcriptional regulator, zinc uptake regulator	1.05	0.004
K06988	fno; 8-hydroxy-5-deazaflavin: NADPH oxidoreductase [EC:1.5.1.40]	1.03	0.005
K08170	norB, norC; MFS transporter, DHA2 family, multidrug resistance protein	1.02	0.001

As for the inferred pathways, 338 KEGG orthologies (KO) were identified across all samples with the most abundant functional pathways belonging to ABC transporters, aminoacyl-tRNA biosynthesis, amino acids and secondary metabolites synthesis, and metabolic pathways which had the highest mean proportional abundance (16.1% in both groups). Similarly, to the inferred genes, there was no significant difference in the most abundant inferred pathways between cases and controls (Figure 5 and Table S4). However, feature selection analysis identified the proteasome pathway, which was not among the top most abundant inferred pathways) to be statistically higher in cases compared to controls (log_2_FC = 1.02, *p* = 0.045).

## Discussion

The design of this first study of the microbial composition of GM for a common metabolic disorder (T2D) provided opportunities to investigate two main questions, namely “what is the distribution of GM in free living urban dwellers in SSA?” and “Does the GM profile differ between cases and controls?.” Similar to previous GM studies conducted in SSA and other human populations (De Filippo et al., 2010, 2017; Schnorr et al., 2014; Morton et al., 2015; Hansen et al., 2019), we found that *Firmicutes* was the single largest microbial community in the control population (Human Microbiome Project Consortium, 2012; Costea et al., 2018). However, in contrast to the other reports including some published African GM studies (De Filippo et al., 2010, 2017; Human Microbiome Project Consortium, 2012; Costea et al., 2018), the relative abundance of *Actinobacteria* (*Coriobacteriaceae, Bifidobacteriaceae*) was approximatively twice (8%) what had been previously reported and considerably higher than *Bacteroidetes*, which is usually the second most abundant phylum in other populations (Human Microbiome Project Consortium, 2012; Costea et al., 2018). Also, in contrast, Bifidobacterial species were not the most represented in the gut of these individuals but rather Coriobacterial species, specifically *Collinsella* was the most abundant. The rank shift between *Actinobacteria* and *Bacteroidetes* in this study sample compared to other populations is remarkable yet not well-understood. *Actinobacteria* plays important physiological roles in the gut including the breakdown of resistant starch, the production of acetate (gut barrier), and the development of the immune system (Binda et al., 2018). The presence of *Actinobacteria* in the gut is influenced by many factors including diet. There is evidence that the abundance of *Collinsella* is a function of the host dietary intake and increases with low-fiber diet (Gomez-Arango et al., 2018). Others have also reported a positive correlation between high-fat diet and *Actinobacteria* abundance (Binda et al., 2018). *Collinsella* is thought to affect both gut leakage and the tight junction proteins in enterocytes, two attributes of metabolic endotoxemia (Gomez-Arango et al., 2018). Potential explanations for the observed differences between the findings of this study and previously published studies of African populations include differences in dietary patterns, urbanization, and changes in diet due to the nutritional transition.

The GM of cases showed distinct patterns compared to that of the controls. First, α-diversity was higher in cases. This finding contrasts with few early reports in T2D where α-diversity is often decreased (Larsen et al., 2010; Lambeth et al., 2015; Dominguez-Bello et al., 2019). This observation may be due to treatment and/or lifestyle changes (e.g., diet, physical activity) that are prescribed following diabetes diagnosis as supported by our analyses that treatment may contribute to increased α-diversity. Second, beta-diversity was associated with T2D but with no distinct clustering when all the phylogenic taxa were used to visualize the data. This suggests that identified correlations are driven by a subset of OTU at lower taxonomic levels as shown by the univariate differential abundance analysis (feature selection).

*Firmicutes* were decreased in cases compared to controls with *Clostridiaceae* and *Peptostreptococcus* explaining most of the difference. A study from Denmark also showed that *Firmicutes* and *Clostridiales* were significantly reduced in T2D (Larsen et al., 2010). In contrast, a study from China showed an increase in the abundance of *Firmicutes* in T2D (Zhang et al., 2013). While conflicting findings regarding the direction of change in the abundance of *Firmicutes* in relation to diabetes has been attributed to difference in ancestry, geographic regions, eating habits, and research methods (Han and Lin, 2014), it should be noted that these two studies had small numbers (fewer than 20 each) of cases.

Several OTUs at the genus level were differentially abundant between cases and controls. These OTUs included *Clostridium* and *Anaerostipes* species (e.g., *Clostridium paraputrificum*, and *Clostridium butyricum*) that were significantly decreased in cases compared to controls. It is known that T2D patients have decreased butyrate-producing bacteria and that reduced butyrate production is associated with insulin resistance (Gao et al., 2009; Qin et al., 2012). In fact, most *Clostridium* and *Anaerostipes* spp. are butyrate producers in the colon (Rivière et al., 2016). The *C. butyricum*, a strictly anaerobic spore-forming bacillus, is a common human and animal gut commensal bacterium that produces a high amount of butyrate. Certain *C. butyricum* strains have been shown to have probiotic properties and are used as probiotics in Asia (Cassir et al., 2016). In murine study, a daily oral gavage with *C. butyricum* improved glycemic indexes (fasting glucose, glucose tolerance, insulin tolerance, GLP-1, and insulin secretion), and decreased blood lipids and inflammatory tone providing early evidence for the anti-diabetic effect of *C. butyricum* (Jia et al., 2017).

Our study also found *Desulfovibrio piger, a* sulfate-reducing spp., to be enriched in T2D. *Desulfovibrionaceae* were significantly abundant in animal model of metabolic syndrome and in animals on high-fat diet (Zhang et al., 2010). Sulfate-reducing spp. produce hydrogen sulfide (H2S), an essential signal transmitter that influence several biological systems including endocrine, cardiovascular, and nervous systems. H2S directly activates the secretion of glucagon-like peptide−1 (GLP-1) and increasing sulfate-reducing spp. in mice using a prebiotic diet led to enhanced GLP-1 secretion, enhanced insulin secretion, improved glucose tolerance, and reduced feeding (Pichette et al., 2017). Interestingly, it has been shown that metformin induce GLP-1 secretion (Sharma and Tripathi, 2019), but the relationship between diabetes treatment e.g., metformin and increased abundance of *Desulfovibrionaceae* is currently unclear. In this study, we did not find any significant association between metformin treatment and the relative abundance of *Desulfovibrionaceae*, an observation that could be due to small sample size. Therefore, it will be important to determine if treatment plays a role in the increase of sulfate-reducing spp. or if the increased in sulfate-reducing spp. as seen in our study is an intrinsic part of the pathogenesis of T2D. In contrast to the studies that found increased opportunistic pathogens (e.g., *Clostridium hatheway, Bacteroides caccae, Escherichia Coli, and Eggerthella lenta*) in diabetes or in mucin-degrading spp. (e.g., Akkermansia) in metformin-treated diabetics (Qin et al., 2012), we did not find any evidence of such enrichment in this study. However, we found that metformin may contribute to the increase of the mean relative abundance of *Verrumicrobiaceae* which includes Akkermansia spp.

We inferred genes and pathways abundance that may be related to the observed compositional changes. Piphillin analysis revealed an enrichment in proteasome pathway in diabetic patients compared to controls; decreased proteasome activity has been implicated in T2D pathology through apoptosis of beta-pancreatic cells in glucotoxic environment in murine models. However, GLP-1-receptor agonist Exendin-4 preserves proteasome activity from the deleterious effects of chronic high glucose (Broca et al., 2014). In light to this later evidence and the relationship between *Desulfovibrionaceae* and the activation of GLP-1 via H2S as discussed above, one can hypothesize that the increased in sulfate-reducing spp. in these diabetic patients may play a role in preserving proteasome activity.

The strengths of this study include the population studied; that is a sub-Saharan urban and adult sample which contrast with previous GM African studies in rural, hunter-gatherer, or pediatric cohorts. Additionally, the design of the study (case-control) allowed us to simultaneously investigate the microbial composition in controls as a proxy of the population and to conduct a comparative microbial study between normoglycemic individuals (controls) and individuals with T2D (cases). One limitation of the study is the inability to properly evaluate the bi-directional relationship between diabetes, diabetes treatment and microbial composition. This was due to the cross-sectional study design which would not allow for inference of longitudinal relationships. In addition, the small number of cases that had no treatment (only 3) and in some treatment groups meant low statistical power to evaluate the potential effect of a specific medication (e.g., SU-only) on GM in this population.

In conclusion, we have shown that non-T2D adults living in a Nigerian city have a characteristic microbial composition that is mainly composed of *Firmicutes* (*Clostridiales*) and *Actinobacteria* (~90%). The GM of cases have a bacterial signature consisting of increased sulfate-reducing spp. *Desulfovibrio piger, Prevotella, Peptostreptococcus, and Eubacterium* and is characterized by a moderate dysbiosis which features a decrease in *Firmicutes*. We also confirmed some previously reported findings such as the decrease in butyrate-producing bacteria seen in diabetes in other populations. Our findings illustrate the importance of studying the microbiome of all human populations. Many factors affect microbial diversity and composition in health and disease,—including genetic background, diet, travel, and co-existence of other microorganisms. Therefore, a better understanding of the relationship between these different factors in developing countries, where the prevalence of cardio-metabolic diseases is steadily increasing, remains critical for a more comprehensive identification of risk factors and in developing preventive strategies.

## Data Availability Statement

Sequence data are available from SRA BioProject PRJNA607849 (http://www.ncbi.nlm.nih.gov/bioproject/607849).

## Ethics Statement

The study was performed in accordance with relevant guidelines and regulations and approved by the National Health Research Ethics of Nigeria and the National Institutes of Health Institutional Review Boards.

## Author Contributions

AD designed the study, analyzed data, interpreted the results, and drafted the manuscript. AA designed the study, analyzed data, reviewed the results, and edited the manuscript. JZ performed data cleaning and management. LL managed samples and assisted with laboratory procedures. SA contributed to study design, did study implementation, and collected clinical data. CA contributed to study design, study implementation, and reviewed the manuscript. CR conceived of the study, did study design, provided the resources to conduct the study, and reviewed the manuscript.

### Conflict of Interest

The authors declare that the research was conducted in the absence of any commercial or financial relationships that could be construed as a potential conflict of interest.
