# Supplementary material for: Gut Microbiome Profiles Are Associated With Type 2 Diabetes in Urban Africans
Source: Front Cell Infect Microbiol. 2020 Feb 25;10:63. doi: 10.3389/fcimb.2020.00063 (PMC7052266; doi:10.3389/fcimb.2020.00063)
Supplement: Supplementary file 1 [file Data_Sheet_1.PDF]

**Supplemental Material**

# Gut microbiome profiles are associated with Type 2 Diabetes in Urban Africans

Ayo P Doumatey*[1], Adebowale Adeyemo*[1], Jie Zhou [1], Lin Lei [1], Sally N Adebamowo [2,3], Clement Adebamowo [2-4], Charles N Rotimi [1]

[1]Center for Research on Genomics and Global Health, National Human Genome Research Institute, National Institutes of Health, Bethesda, MD

[2]Department of Epidemiology and Public Health and Greenebaum Comprehensive Cancer Center, University of Maryland School of Medicine, Baltimore, MD

[3]Center for Bioethics and Research, Ibadan, Nigeria

[4]Institute of Human Virology, University of Maryland School of Medicine, Baltimore, MD

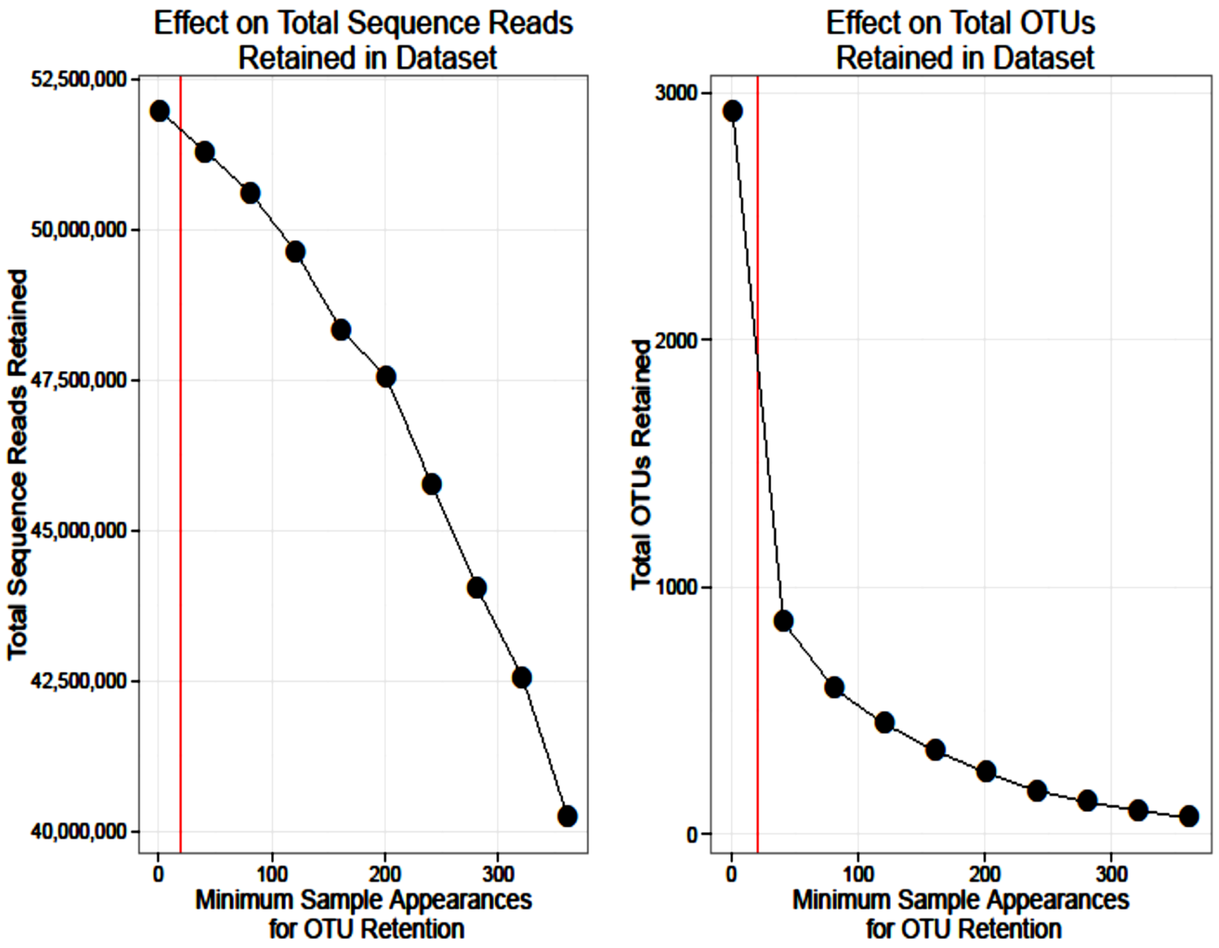


**Figure S1. Independent filtering**

Removal of spurious OTUs was completed by independent filtering. OTUs seen at least once within 5% of the data were retained.

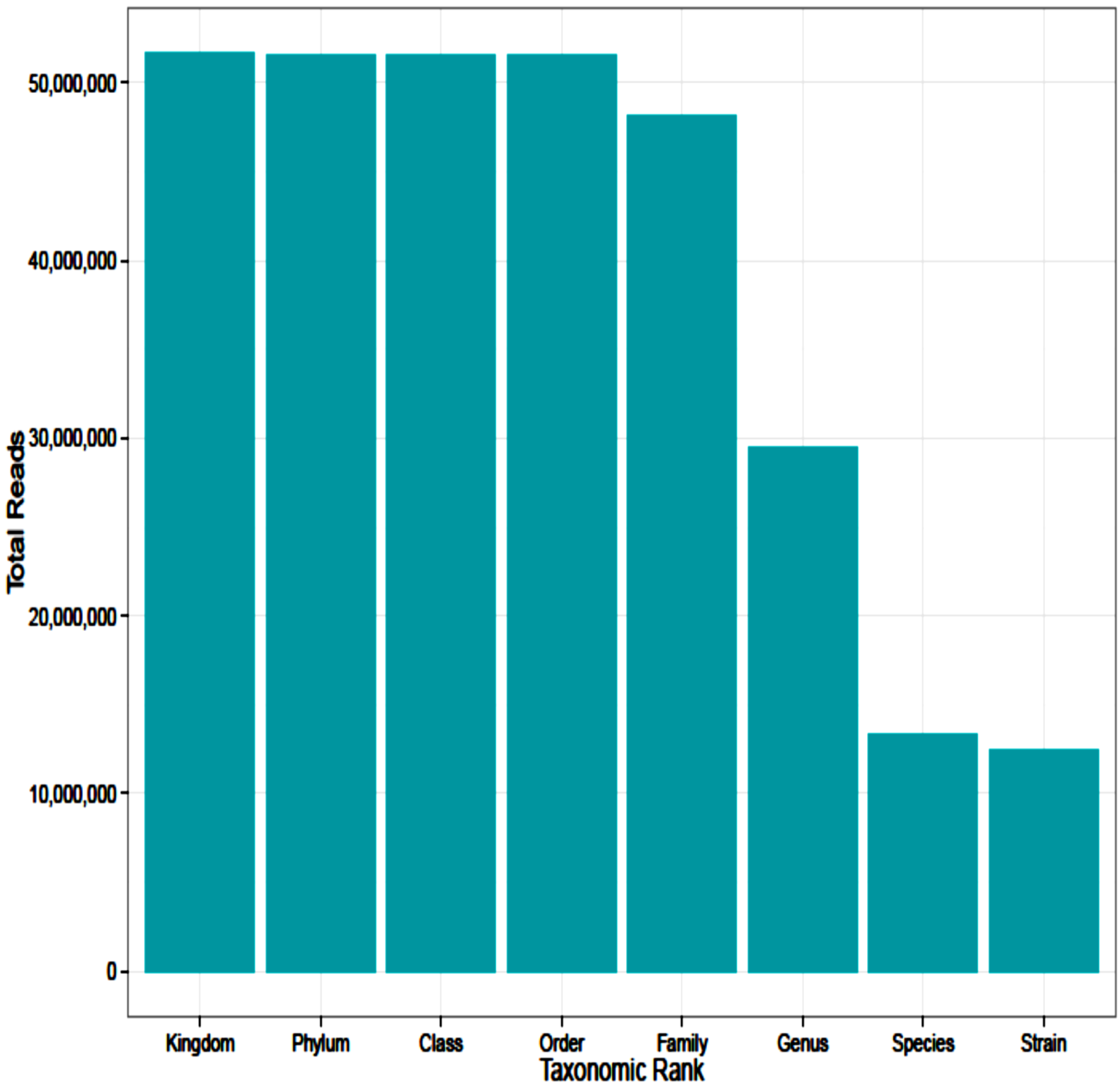


**Figure S2. Classification of sequences**

Bar plot summarizes taxonomic classification success of sequences of all the samples. Kingdom to order ~100% classified. Family, about 93% were classified; Genus, about 57% classified, species and strain 25.7% and 24.2% respectively were classified.

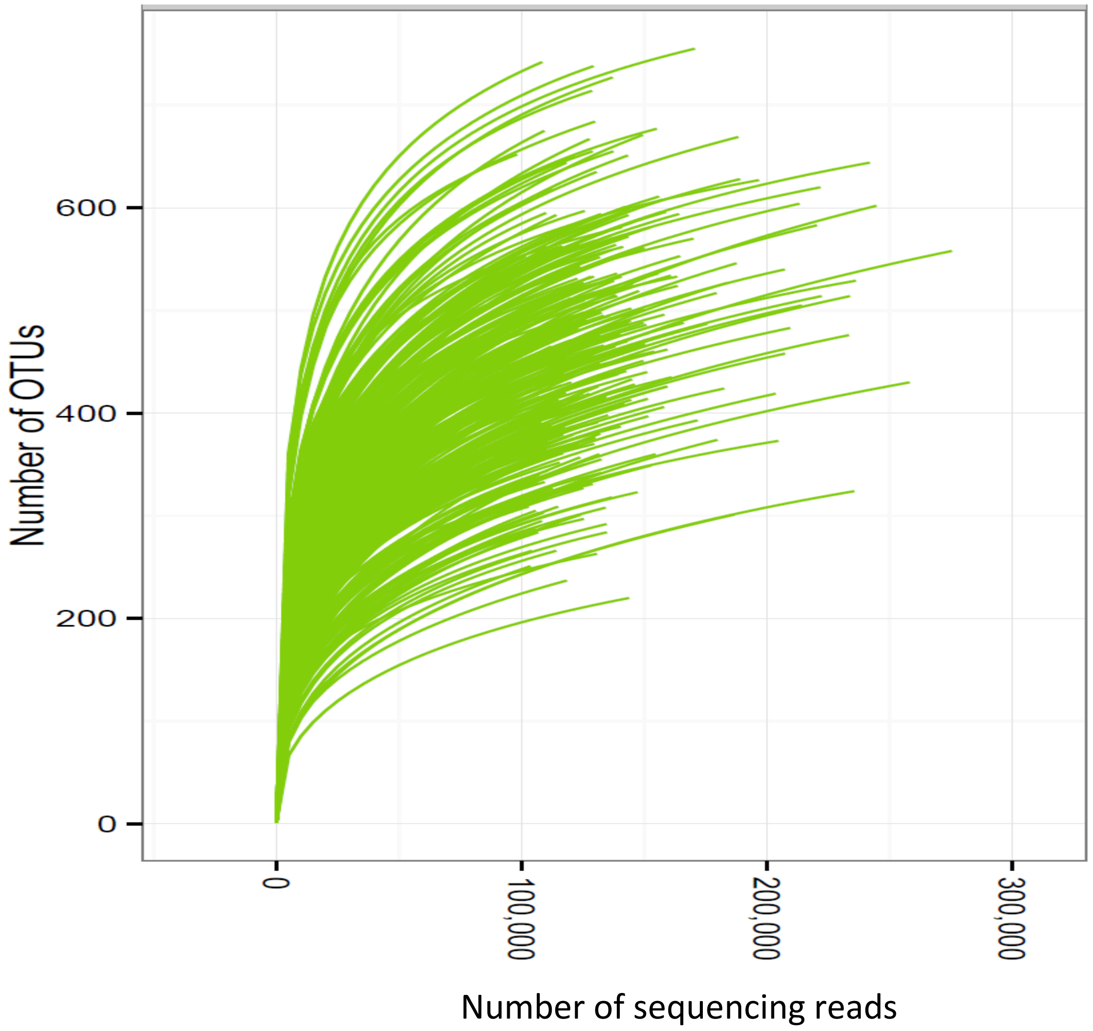


**Figure S3. Rarefaction curves**

Average number of OTUs detected vs. sequencing library size. A curve approaching a horizontal slope is nearly saturated with few new OTUs unidentified. A curve with a steep slope has not been sequenced to saturation and additional OTUs can be discovered with deeper sequencing.

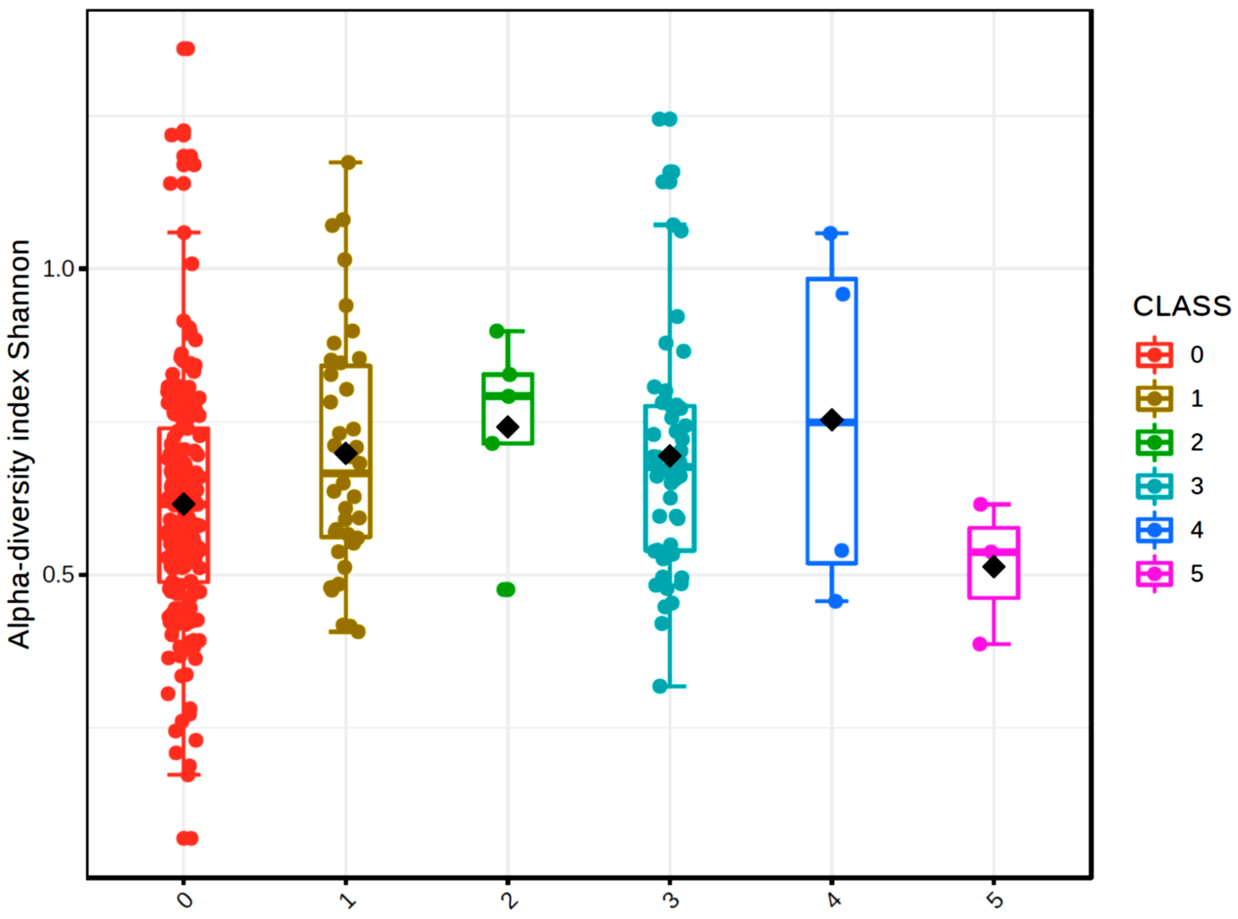


**Figure S4. Alpha- diversity estimate (Shannon Index) by diabetes treatment classes**

Controls (0), Metformin-only (1), Sulfonylurea-only (2), Metformin+ Sulfonylurea (3), Other combinations of antidiabetic medicines (4), Untreated diabetic patients (5)
Black diamond represents median Shannon Index

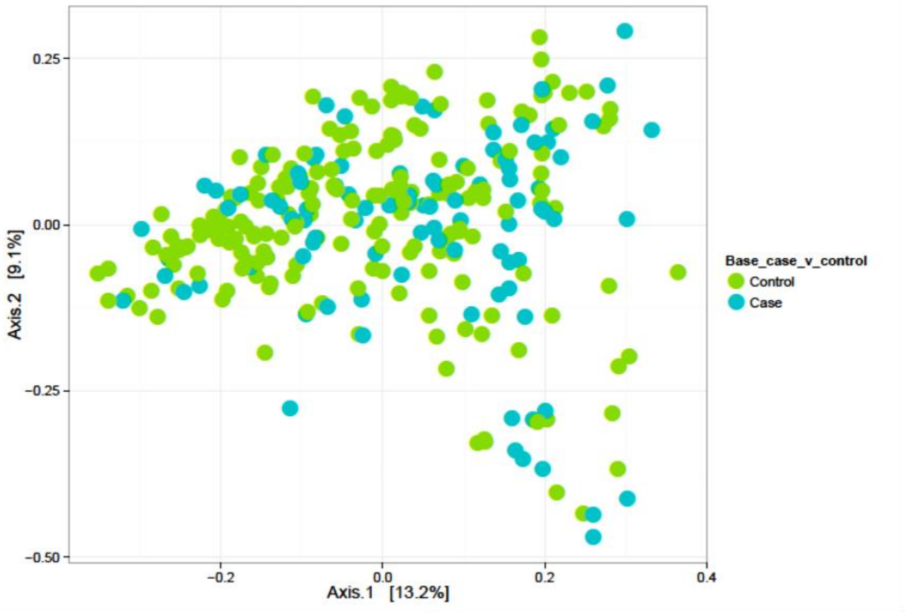


**Figure S5a. Weighted ordination**

Dimension reduction on the Bray-Curtis distance between microbiome samples from cases and controls using the PCoA ordination method. The first two ordination axes explained 22.3% of the sample variation.

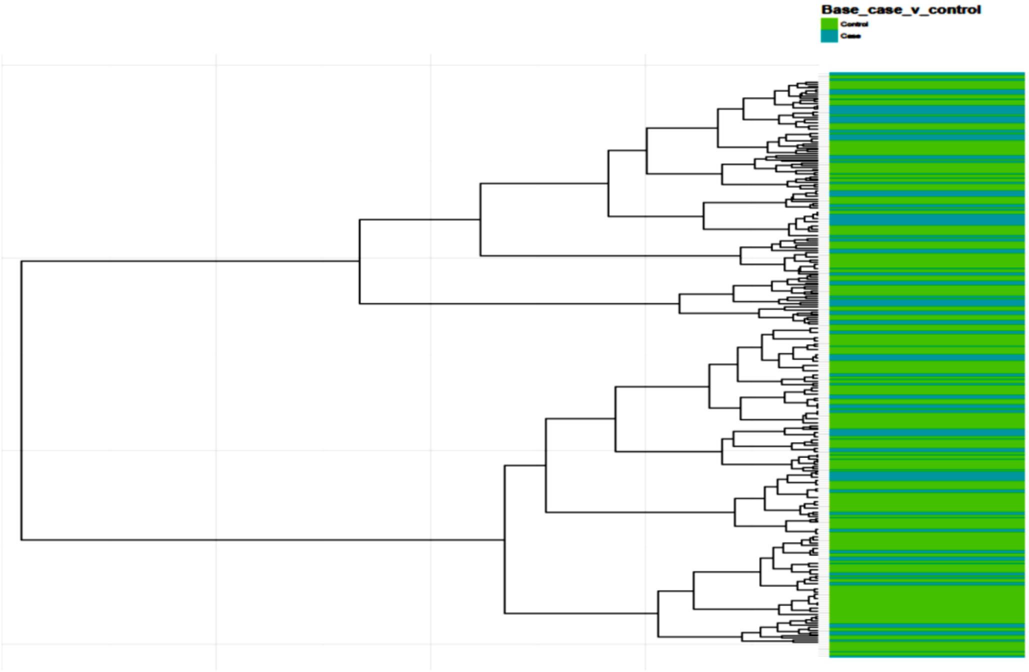


**Figure S5b. Hierarchical clustering for cases and controls**
Clustered by the Ward's method and Bray-Curtis distance

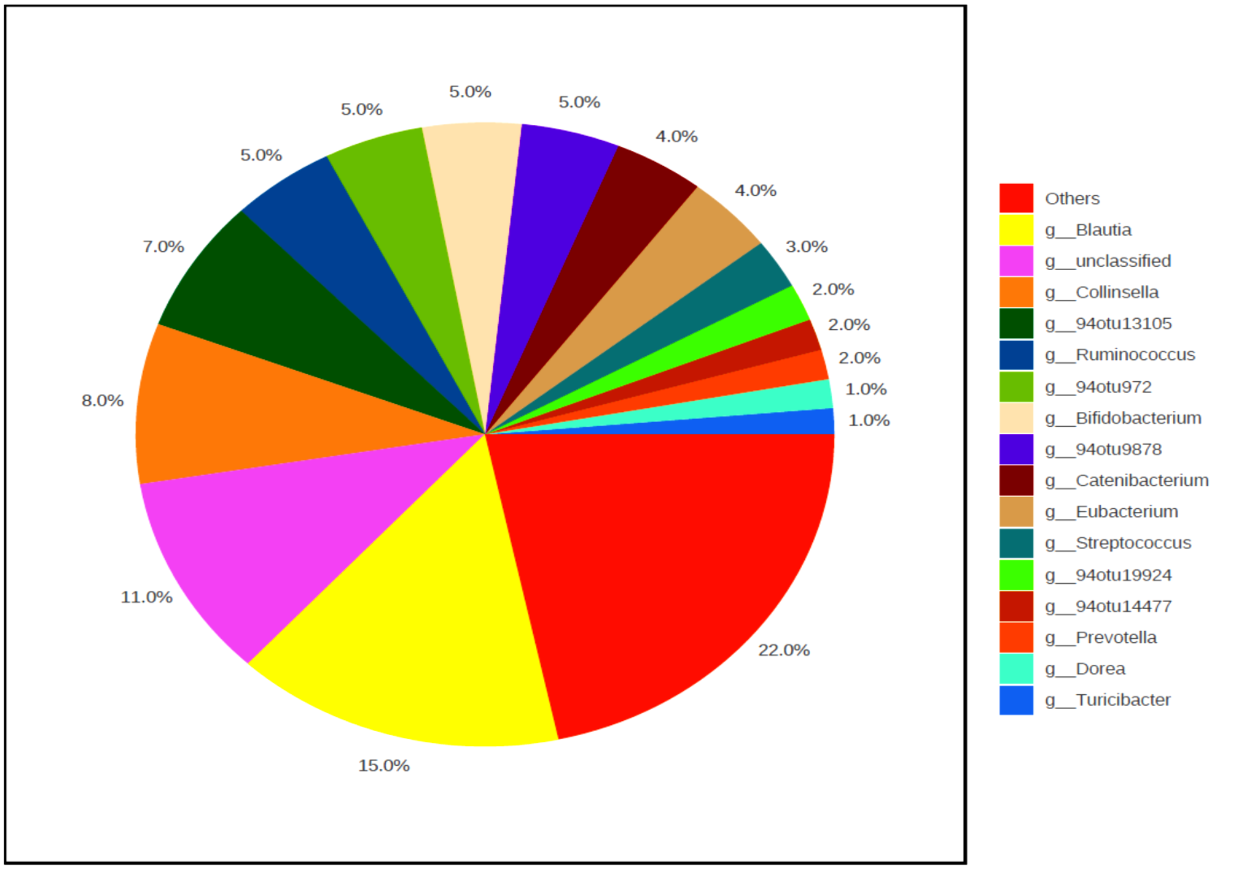


**Figure S6. Pie-chart of genera identified in controls**

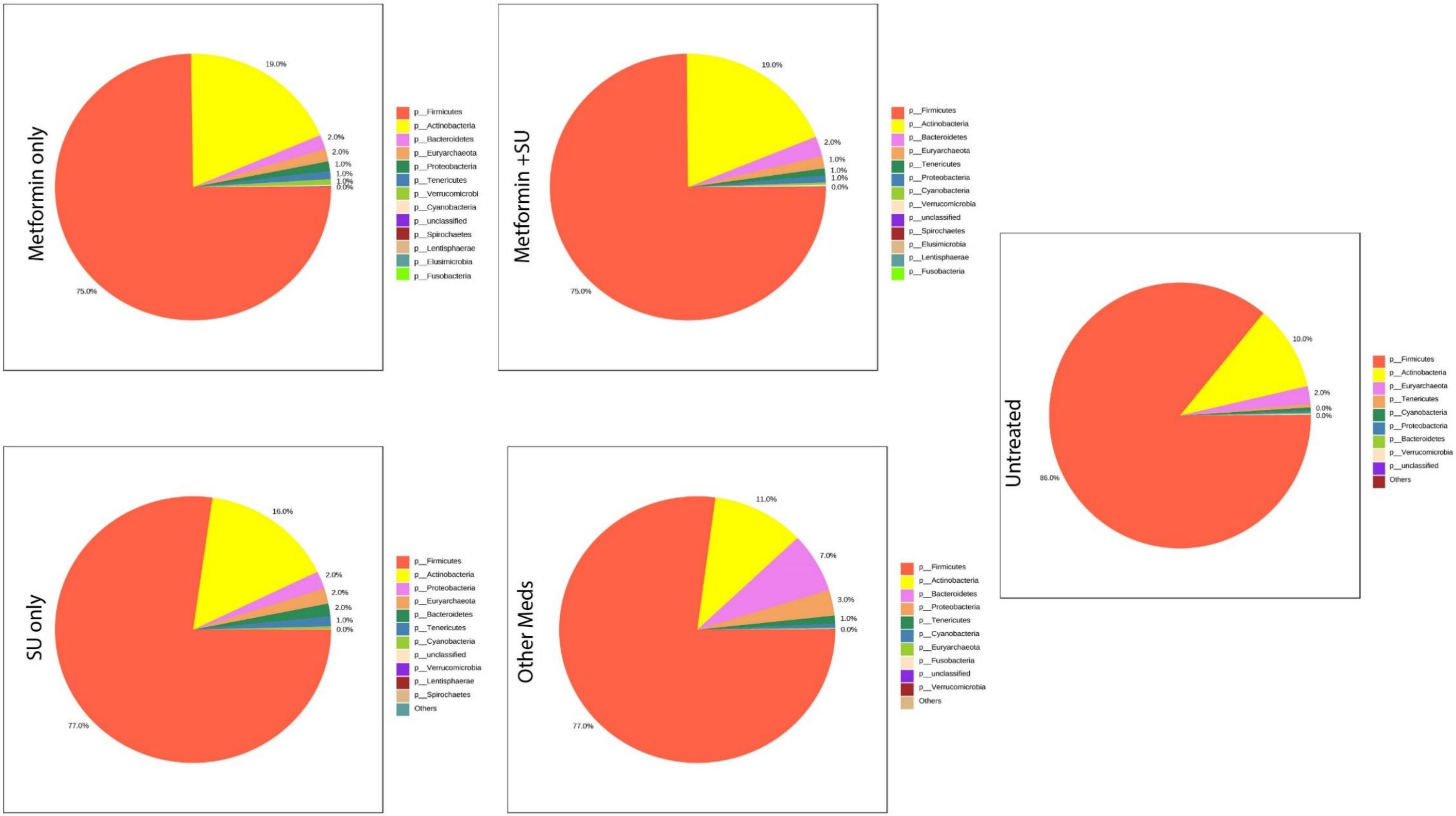


**Figure S7. Pie-charts of most represented phyla in cases by anti-diabetic treatment**

SU= Sulfonylurea; Other meds= other combinations of antidiabetic medicines

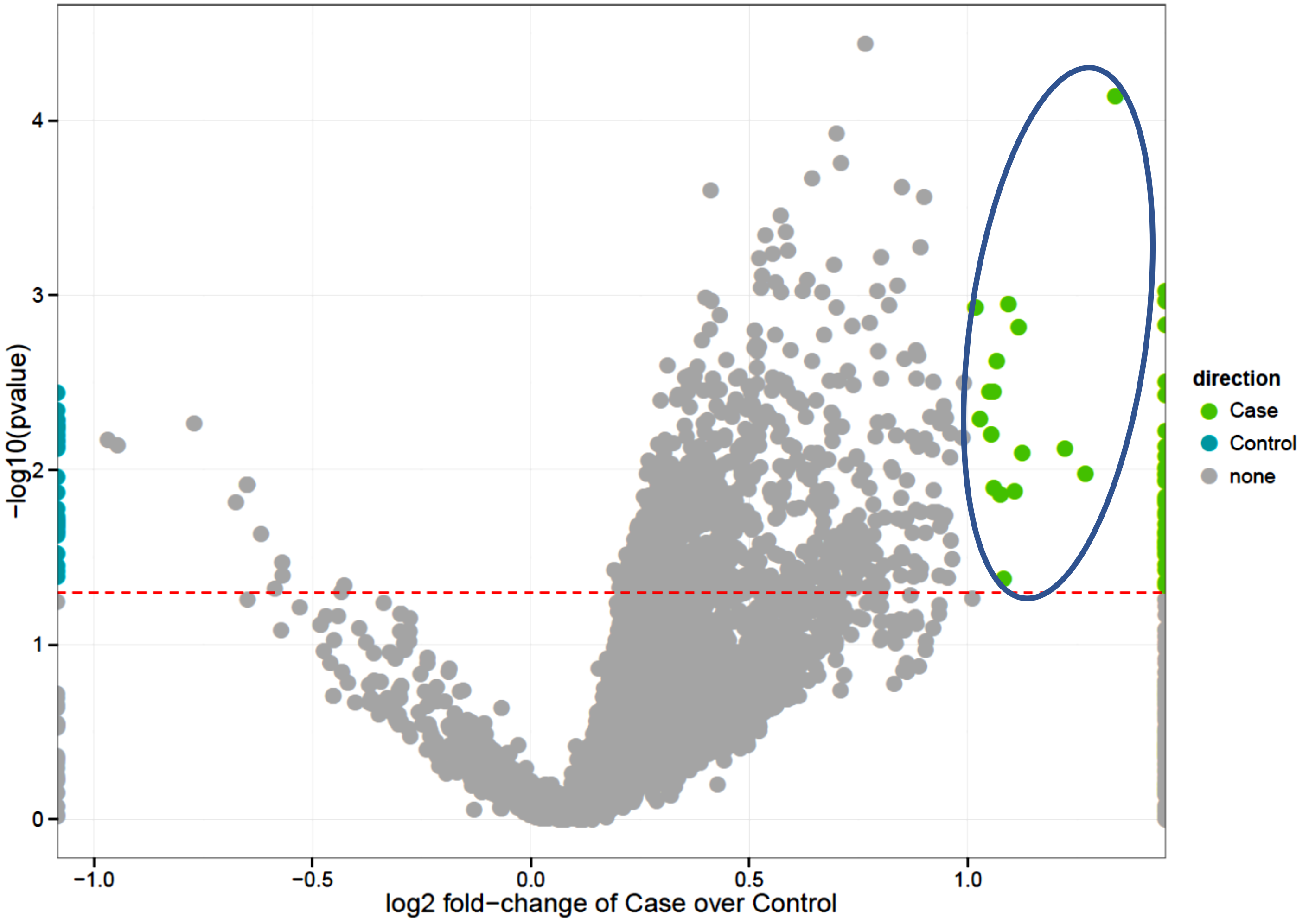


**Figure S8. Feature Selection on Inferred Genes for cases vs controls**

Red dotted line represents alpha at 0.05. Points above alpha line have unadjusted p<0.05 and below unadjusted p>0.05. Features were considered significant if adjusted p-value was <0.05, and absolute log2 fold-change was greater than 1. Points at either end of x-axis indicate infinite log-2 fold-change (the other group's mean abundance is 0). Features with significant unadjusted p-values that met the log2 fold-change threshold are highlighted in color and circled in blue – a total of 16 genes.

**Table S1. Covariate significance analysis for alpha-diversity and beta-diversity in the gut microbiome study**

| | Shannon Index | | OTU richness | | Beta-diversity PERMANOVA |
|---|---|---|---|---|---|
| **Characteristic** | Rho | P value | Rho | P value | P value |
| **Gender** | 1.689 | 0.1937 | 0.763 | 0.3824 | 0.032 |
| **Age** | 0.0699 | 0.3343 | 0.1030 | 0.1540 | 0.61 |
| **BMI** | 0.0061 | 0.9332 | -0.0138 | 0.8494 | 0.521 |
| **Waist circumference** | 0.0719 | 0.3206 | 0.0948 | 0.1897 | 0.492 |
| **Percent fat mass** | -0.0234 | 0.7462 | -0.0106 | 0.8833 | 0.863 |

**Table S2. Effect of metformin assessed by regression analyses**

**Table S2-A. Effect of metformin at phylum level (non-parametric regression)**

| | **Unadjusted model** | | | **Adjusted model*** | | |
|---|---|---|---|---|---|---|
| **RA of the most abundant Phyla** | **Observed estimate (se)#** | **p-value** | **R-squared** | **Observed estimate (se)#** | **p-value** | **R-squared** |
| **Firmicutes** | -4.86 (1.71) | **0.004** | 0.032 | -4.23 (4.66) | **0.36** | 0.35 |
| **Actinobacteria** | .76 (0.42) | 0.07 | 0.011 | 3.73 (3.36) | 0.27 | 0.29 |
| **Bacteroidetes** | 0.26 (0.13) | **0.05** | 0.0036 | 0.39 (0.77) | 0.51 | 0.27 |
| **Proteobacteria** | -0.014 (0.06) | 0.82 | 0.0002 | -0.27 (0.76) | 0.725 | 0.51 |
| **Euryarchaeota** | -0.009 (0.049) | 0.86 | 0.0001 | -0.03 (0.58) | 0.96 | 0.37 |
| **Tenericutes** | 0.011 (0.02) | 0.56 | 0.0012 | -0.027 (0.35) | 0.94 | 0.29 |
| **Verrucomicrobia** | 0.06 (0.05) | 0.163 | 0.026 | 0.44 (0.55) | 0.42 | 0.30 |
| **Cyanobacteria** | -.0026 (0.01) | 0.79 | 0.0002 | 0.017 (0.17) | 0.91 | 0.18 |

# estimates for metformin effect in the model; *adjustment for age, sex, and BMI
Se: standard error

**Table S2-B. Effect of metformin at Family level (Poisson regression)**

| | **Unadjusted model** | | | **Adjusted model*** | | |
|---|---|---|---|---|---|---|
| | **Observed Coef. (se)#** | **p-value** | **R-squared** | **Observed Coef. (se)#** | **p-value** | **R-squared** |
| **Verrucomicrobiaceae** | 2.14 (1.00) | 0.03 | **0.02** | 2.87 (1.68) | **0.088** | 0.122 |
| **Lachnospiraceae** | -0.08 (0.19) | 0.66 | 0.002 | -0.062 (0.17) | 0.73 | 0.011 |
| **Coriobacteriaceae** | 0.18 (0.15) | 0.23 | 0.0040 | 0.22 (0.20) | 0.27 | 0.022 |
| **Erysipelotrichaceae** | -0.03 (0.27) | 0.91 | 0.0001 | -0.099 (0.19) | 0.61 | 0.018 |
| **Clostridiaceae** | -0.39 (0.27) | 0.14 | 0.01 | -0.33 (0.33) | 0.326 | 0.026 |
| **Ruminococcaceae** | -0.037 (0.41) | 0.93 | 0.0001 | -0.048 (0.45) | 0.91 | 0.063 |
| **Peptostreptococcaceae** | -0.29 (0.47) | 0.54 | 0.060 | -0.34 (0.45) | 0.44 | 0.012 |
| **Bifidobacteriaceae** | 0.50 (1.10) | 0.65 | 0.0075 | 0.53 (0.85) | 0.53 | 0.082 |
| **Desulfovibrionaceae** | 1.51 (1.18) | 0.200 | 0.011 | 1.46 (1.23) | 0.23 | 0.023 |

**Table S3. Mean and standard deviation (SD) percent relative abundances of the top 8 genes**

| Gene Description | Controls | Cases |
|---|---|---|
| ABC-2A; ABC-2 type transport system ATP-binding protein | 0.506 (0.134) | 0.505 (0.139) |
| ABCB-BAC; ATP-binding cassette, subfamily B, bacterial | 0.627 (0.127) | 0.606 (0.145) |
| ABC.CD.A; putative ABC transport system ATP-binding protein | 0.659 (0.149) | 0.656 (0.165) |
| LacI, galR; LacI family transcriptional regulator | 0.407 (0.147) | 0.404 (0.148) |
| Others | 96.1 (0.53) | 96.2 (0.618) |
| rpoE; RNA polymerase sigma-70 factor, ECF subfamily | 0.403 (0.19) | 0.391 (0.154) |
| tRNA-Arg; tRNA Arg | 0.356 (0.0796) | 0.342 (0.0666) |
| tRNA-Leu; tRNA Leu | 0.316 (0.0688) | 0.321 (0.0549) |
| ABC.CD.P; putative ABC transport system permease protein | 0.613 (0.137) | 0.608 (0.151) |

**Table S4. Mean and standard deviation (SD) percent relative abundances of the top 8 pathways**

| Pathway | Controls | Cases |
|---|---|---|
| ABC transporters | 2.79 (0.447) | 2.77 (0.392) |
| Aminoacyl-tRNA biosynthesis | 2.86 (0.271) | 2.90 (0.227) |
| Biosynthesis of amino acids | 4.25 (0.395) | 4.3 (0.323) |
| Biosynthesis of antibiotics | 5.56 (0.312) | 5.61 (0.25) |
| Biosynthesis of secondary metabolites | 7.31 (0.255) | 7.31 (0.26) |
| Carbon metabolism | 2.5 (0.174) | 2.55 (0.255) |
| Metabolic pathways | 16.1 (0.228) | 16.1 (0.262) |
| Microbial metabolism in diverse environments | 4.52 (0.376) | 4.55 (0.457) |
| Others | 54.1 (0.841) | 53.9 (0.658) |
